# Beyond gene-disease validity: capturing structured data on inheritance, allelic requirement, disease-relevant variant classes, and disease mechanism for inherited cardiac conditions

**DOI:** 10.1186/s13073-023-01246-8

**Published:** 2023-10-23

**Authors:** Katherine S. Josephs, Angharad M. Roberts, Pantazis Theotokis, Roddy Walsh, Philip J. Ostrowski, Matthew Edwards, Andrew Fleming, Courtney Thaxton, Jason D. Roberts, Melanie Care, Wojciech Zareba, Arnon Adler, Amy C. Sturm, Rafik Tadros, Valeria Novelli, Emma Owens, Lucas Bronicki, Olga Jarinova, Bert Callewaert, Stacey Peters, Tom Lumbers, Elizabeth Jordan, Babken Asatryan, Neesha Krishnan, Ray E. Hershberger, C. Anwar A. Chahal, Andrew P. Landstrom, Cynthia James, Elizabeth M. McNally, Daniel P. Judge, Peter van Tintelen, Arthur Wilde, Michael Gollob, Jodie Ingles, James S. Ware

**Affiliations:** 1 National Heart and Lung Institute, Imperial College London, Du Cane Road, London, W12 0NN UK; 2 Royal Brompton and Harefield Hospitals, Guy’s and St Thomas’ NHS Foundation Trust, London, UK; 3 Great Ormond Street Hospital, NHS Foundation Trust, London, UK; 4 Amsterdam University Medical Centre, University of Amsterdam, Heart Center, Department of Experimental Cardiology, Amsterdam Cardiovascular Sciences, Amsterdam, The Netherlands; 5 Clinical Genetics & Genomics Lab, Royal Brompton and Harefield Hospitals, Guy’s and St Thomas’ NHS Foundation Trust, London, UK; 6 Department of Genetics, University of North Carolina at Chapel Hill, Chapel Hill, NC USA; 7 Population Health Research Institute, McMaster University, and Hamilton Health Sciences, Hamilton, Ontario Canada; 8 Department of Molecular Genetics, University of Toronto, Toronto, Canada; 9 Division of Cardiology, Toronto General Hospital, Toronto, Canada; 10 Clinical Cardiovascular Research Center, University of Rochester, Rochester, NY USA; 11 Division of Cardiology, Peter Munk Cardiac Centre, University Health Network and Department of Medicine, University of Toronto, Toronto, Ontario Canada; 12 23andMe, Genomic Health, Sunnyvale, CA USA; 13 Cardiovascular Genetics Center, Montreal Heart Institute, and Faculty of Medicine, Université de Montréal, Montreal, Canada; 14 Unit of Immunology and Functional Genomics, Centro Cardiologico Monzino IRCCS, Milano, Italy; 15 Department of Pathology and Laboratory Medicine, University of Ottawa, Ottawa, Ontario Canada; 16 Department of Genetics, CHEO, Ottawa, Ontario Canada; 17 Center for Medical Genetics, Ghent University Hospital, Ghent, Belgium; 18 Department of Biomolecular Medicine, Ghent University, Ghent, Belgium; 19 Department of Cardiology and Genomic Medicine, Royal Melbourne Hospital, Melbourne, Australia; 20 University of Melbourne, Melbourne, Australia; 21 Barts Health & University College London Hospitals NHS Trusts, London, UK; 22 Institute of Health Informatics, University College London, London, UK; 23 Divisions of Human Genetics and Cardiovascular Medicine, The Ohio State University, Columbus, OH USA; 24 Department of Cardiology, Inselspital, Bern University Hospital, University of Bern, Bern, Switzerland; 25 Division of Cardiology, Department of Medicine, Johns Hopkins University School of Medicine, Baltimore, MD USA; 26 Centre for Population Genomics, Garvan Institute of Medical Research, and UNSW Sydney, Sydney, Australia; 27 Center for Inherited Cardiovascular Diseases, WellSpan Health, Lancaster, PA USA; 28 Cardiac Electrophysiology and Inherited Cardiovascular Diseases, Cardiovascular Division, Hospital of the University of Pennsylvania, Philadelphia, PA USA; 29 Department of Cardiovascular Medicine, Mayo Clinic, Rochester, MN USA; 30 Barts Heart Centre, St Bartholomew’s Hospital, Barts Health NHS Trust, London, UK; 31 Department of Pediatrics and Cell Biology, Duke University School of Medicine, Durham, NC USA; 32 Johns Hopkins Center for Inherited Heart Diseases, Department of Medicine, Johns Hopkins University, Baltimore, MD USA; 33 Center for Genetic Medicine, Dept of Medicine (Cardiology), Northwestern University Feinberg School of Medicine, Chicago, IL USA; 34 Medical University of South Carolina, Charleston, SC USA; 35 Department of Genetics, University Medical Center Utrecht, Utrecht, the Netherlands; 36 Department of Cardiology, Amsterdam UMC location University of Amsterdam, Meibergdreef 9, Amsterdam, the Netherlands; 37 Amsterdam Cardiovascular Sciences, Heart Failure and Arrhythmias, Amsterdam UMC location University of Amsterdam, Amsterdam, the Netherlands; 38 Inherited Arrhythmia and Cardiomyopathy Program, Division of Cardiology, University of Toronto, Toronto, ON Canada; 39 MRC London Institute of Medical Sciences, Imperial College London, London, UK

**Keywords:** Inherited cardiac conditions, Inheritance, Allelic requirement, Disease mechanism, Gene curation, Genomic variant filtering, Variant interpretation, Variant classification

## Abstract

**Background:**

As the availability of genomic testing grows, variant interpretation will increasingly be performed by genomic generalists, rather than domain-specific experts. Demand is rising for laboratories to accurately classify variants in inherited cardiac condition (ICC) genes, including secondary findings.

**Methods:**

We analyse evidence for inheritance patterns, allelic requirement, disease mechanism and disease-relevant variant classes for 65 ClinGen-curated ICC gene-disease pairs. We present this information for the first time in a structured dataset, CardiacG2P, and assess application in genomic variant filtering.

**Results:**

For 36/65 gene-disease pairs, loss of function is not an established disease mechanism, and protein truncating variants are not known to be pathogenic. Using the CardiacG2P dataset as an initial variant filter allows for efficient variant prioritisation whilst maintaining a high sensitivity for retaining pathogenic variants compared with two other variant filtering approaches.

**Conclusions:**

Access to evidence-based structured data representing disease mechanism and allelic requirement aids variant filtering and analysis and is a pre-requisite for scalable genomic testing.

**Supplementary Information:**

The online version contains supplementary material available at 10.1186/s13073-023-01246-8.

## Background

Inherited cardiac conditions (ICCs) are a group of disorders that share the potential for devastating outcomes, including heart failure and sudden cardiac death at a young age.

Early diagnosis is vital and allows prompt treatment, risk stratification, and primary prevention for sudden cardiac arrest in high-risk individuals. Genetic testing is a routine part of evaluation and can aid diagnosis and alter clinical management [1–3].

The scope of genetic testing for ICC-associated genes is growing. In addition to patients undergoing evaluation for confirmed or suspected disease, opportunistic screening for secondary findings is increasing as more patients undergo exome (ES) or genome sequencing (GS) in diverse clinical settings or via consumer-initiated testing. A recent statement by the American Heart Association (AHA) highlights the challenges in interpreting incidental and secondary findings [4]. There are 47 of 90 medically actionable gene-disease pairs on the American College of Medical Genetics and Genomics Secondary Findings list (ACMG SF V3.1) [5] related to cardiovascular (CV) disease. The ACMG recommends that these genes are analysed whenever clinical ES or GS is performed and that pathogenic or likely pathogenic (P/LP) variants are reported back to patients. Therefore, many laboratories, regardless of their expertise, will soon need the capability to rapidly interpret variants in CV genes. This creates the potential for variant misclassification and/or poor communication of the interpretation of secondary findings to clinicians which could have significant downstream effects on patients and their families [6].

As access to sequencing and sharing of genomic data has improved, the number of genes and variants reported to be associated with any given disease has grown. Bioinformatic filtering pipelines often prioritise protein truncating variants that are indeed enriched for disease-causing variants in aggregate, but may not be pathogenic if loss of function (LoF) is not a mechanism for the relevant disease. At best, this results in time-consuming false positives and, at worst, can lead to misinterpretation of genomic test results. For ICCs, incomplete penetrance, genetic heterogeneity, oligogenic and modifying variants, overlapping phenotypes, and different disease mechanisms make variant interpretation particularly challenging.

There are international efforts underway to re-evaluate the validity of previously published gene-disease relationships. The Gene Curation Coalition (GenCC) [7] is a consortium of parties engaged in gene curation, and theGenCC.org (https://search.thegencc.org/) [8] is a harmonised repository of curated gene-disease relationships from many groups. Having established a robust gene-disease relationship, clinical interpretation of variation within a disease gene is critically dependent on an understanding of the allelic requirement for the disease, and of the mechanism of pathogenicity and disease-relevant variant classes. This data has not previously been consistently available in a structured format for variant prioritisation.

Here, we have analysed the inheritance, allelic requirement, disease mechanism, and disease-relevant variant classes for robust ICC-associated gene-disease pairs using a standardised terminology recently developed by the GenCC [9]. The results of this analysis have been approved by international multidisciplinary expert review panels comprised of scientists and clinicians with expertise in ICCs. Structured data sets with this type of information do not exist currently and are shared here and as a publicly available resource, CardiacG2P, to aid in filtering and analysis of ICC genetic variants.

CardiacG2P is an evidence-based dataset hosted on G2P (https://www.ebi.ac.uk/gene2phenotype), an online system set up to establish, curate and distribute datasets for diagnostic variant filtering [10]. Each dataset entry annotates a disease with an allelic requirement, information pertaining to the disease mechanism (represented as a disease-associated variant consequence), and known disease-relevant variant classes at a defined locus. This dataset is compatible with the existing G2P Ensembl Variant Effect Predictor (VEP) [11] plugin to support automated filtering of genomic variants accounting for inheritance pattern and mutational consequence. Other G2P datasets for developmental disorders and ophthalmic conditions have shown this approach can help to discriminate between variants, improving the precision of diagnostic variant filtering [10, 12]. G2P data are also available through the GenCC hub [8]. Here we assess CardiacG2P and show its impact on the efficiency of variant prioritisation.

## Methods

### Analysis of inheritance and disease-associated variant consequences in genes implicated in inherited cardiac conditions

**Fig. 1:**
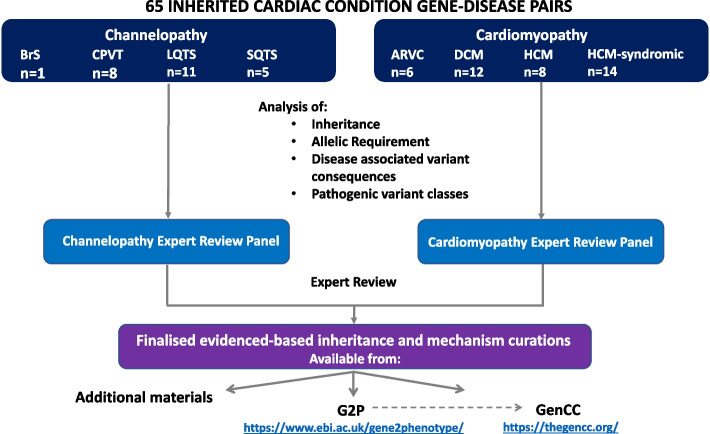
Flow chart depicting the analysis of inheritance and disease mechanism in established inherited cardiac genes. A structured representation of the resulting data is available in the Additional files 2 and 3 and also through G2P (https://www.ebi.ac.uk/gene2phenotype/downloads), which is also searchable through the GenCC portal (https://thegencc.org/). ARVC, arrhythmogenic right ventricular cardiomyopathy; BrS, Brugada syndrome; CPVT, catecholaminergic polymorphic ventricular tachycardia; DCM, dilated cardiomyopathy; G2P, gene2phenotype; GenCC, Gene Curation Coalition; HCM, hypertrophic cardiomyopathy; LQTS, long QT syndrome; SQTS, short QT syndrome

We analysed evidence to determine the inheritance pattern, allelic requirement, disease mechanism and disease-relevant variant classes for 65 gene-disease pairs for major ICCs (Fig. 1). We analysed genes classified with “Definitive” or “Strong” evidence by The Clinical Genome Resource (ClinGen) Gene Curation Expert Panels (GCEPs) for seven CV diseases under a Mendelian (monogenic) model (accessed November 2020) [13, 14]: hypertrophic cardiomyopathy (HCM), dilated cardiomyopathy (DCM), arrhythmogenic right ventricular cardiomyopathy (ARVC), long QT syndrome (LQTS), Brugada syndrome (BrS), catecholaminergic polymorphic ventricular tachycardia (CPVT), and short QT syndrome (SQTS) [15–20]. Information on these ClinGen expert panels, membership, and curation activity can be found at www.clinicalgenome.org. For HCM, we included both genes causing typical HCM and also genes associated with syndromic disorders where apparently isolated left ventricular hypertrophy (LVH) may be the presenting feature (genocopies) [19].

Seven channelopathy gene-disease pairs classified by ClinGen as having “Moderate” strength of evidence for monogenic disease are included (*CALM1-*CPVT*, CALM2-*CPVT*, CALM3-*CPVT*, CASQ2-*CPVT*, KCNE1-*JLN*, SLC4A3*-SQTS, *KCNJ2*-SQTS), following discussion with the channelopathy expert review panel for this project, and where there was sufficient data to adjudicate the required fields. *SLC22A5* was also evaluated as a phenotypic mimic of SQTS: although it is classified as “Disputed” by ClinGen Short QT GCEP in relation to true SQTS, it is definitively associated with systemic primary carnitine deficiency disease, which can present similarly to SQTS and might reasonably be included in gene panels for diagnostic assessment of patients presenting with this phenotype. See Tables 1 and 2 and Additional file 3: Table S1 for a complete list of the gene-disease pairs evaluated.

**Table 1:** Structured representation of data from curation of core cardiomyopathy gene-disease pairs (HCM, DCM, ARVC)

**Cardiomyopathy**					
**Gene**	**Gene-disease validity**^**a**^	**Inheritance**	**Allelic requirement**	**Disease-associated variant consequence**	**Variant classes reported with evidence of pathogenicity**
**Hypertrophic cardiomyopathy**					
*ACTC1*	Definitive	AD	Monoallelic autosomal	Altered gene product sequence	Missense; inframe deletion
*MYBPC3*	Definitive	AD^c^	Monoallelic autosomal	Decreased gene product level; altered gene product sequence	Missense; inframe indels; NMD truncating^e^; structural variants (whole exon deletions)
*MYH7*	Definitive	AD^c^	Monoallelic autosomal	Altered gene product sequence	Missense; inframe deletion; stop gained NMD escaping
*MYL2*	Definitive	AD	Monoallelic autosomal	Altered gene product sequence	Missense
*MYL3*	Definitive	AD	Monoallelic autosomal	Altered gene product sequence	Missense
*TNNI3*	Definitive	AD^c^	Monoallelic autosomal	Altered gene product sequence	Missense; inframe deletion
*TNNT2*	Definitive	AD^c^	Monoallelic autosomal	Altered gene product sequence	Missense; inframe deletion; stop gained NMD escaping; splice donor variant NMD escaping
*TPM1*	Definitive	AD^c^	Monoallelic autosomal	Altered gene product sequence	Missense
**Dilated cardiomyopathy**					
*BAG3*	Definitive	AD^c^	Monoallelic autosomal	Decreased gene product level; altered gene product sequence	Missense; NMD truncating^e^; structural variants (whole exon deletions); copy number variants (whole gene deletion)
*DES*	Definitive	AD^c;d^	Monoallelic autosomal	Altered gene product sequence	Missense; splice acceptor variant NMD escaping
*DSP*	Strong	AD^c^	Monoallelic autosomal	Decreased gene product level; altered gene product sequence	Missense; NMD truncating^e^;
*FLNC*	Definitive	AD^c^	Monoallelic autosomal	Decreased gene product level	NMD truncating^e^
*LMNA*	Definitive	AD^d^	Monoallelic autosomal	Decreased gene product level; altered gene product sequence	Missense; NMD truncating^e^; structural variants (whole exon deletions)
*MYH7*	Definitive	AD^c^	Monoallelic autosomal	Altered gene product sequence	Missense
*RBM20*	Definitive	AD^d^	Monoallelic autosomal	Decreased gene product level; altered gene product sequence	Missense; NMD truncating^e^
*SCN5A*	Definitive	AD^d^	Monoallelic autosomal	Decreased gene product level; altered gene product sequence	Missense; NMD truncating^e^
*TNNC1*	Definitive	AD	Monoallelic autosomal	Altered gene product sequence	Missense
*TNNT2*	Definitive	AD^d^	Monoallelic autosomal	Altered gene product sequence	Missense
*TTN*	Definitive	AD^c^	Monoallelic autosomal	Decreased gene product level; Altered gene product sequence	NMD truncating^e^ (variants must impact exons (PSI > 0.9);Limited repertoire of missense variants established as pathogenic
*PLN* (IC)^b^	Definitive	AD^c^	Monoallelic autosomal	Decreased gene product level; altered gene product sequence	Missense; inframe indels; NMD truncating^e^; structural variants (whole exon deletions)
**Arrhythmogenic right ventricular cardiomyopathy**					
*DSC2*	Definitive	AD; AR^c^	Monoallelic autosomal; biallelic autosomal	Decreased gene product level; altered gene product sequence	Missense; inframe indels; NMD truncating^e^
*DSG2*	Definitive	AD; AR^c^	Monoallelic autosomal; biallelic autosomal	Decreased gene product level; altered gene product sequence	Missense; inframe indels; NMD truncating^e^
*DSP*	Definitive	AD; AR^c^	Monoallelic autosomal; biallelic autosomal	Decreased gene product level; altered gene product sequence	Missense; inframe indels; NMD truncating^e^
*PKP2*	Definitive	AD^c^; AR	Monoallelic autosomal; Biallelic autosomal	Decreased gene product level; altered gene product sequence	Missense; inframe indels; NMD truncating^e^; structural variants
*TMEM43*	Definitive	AD	Monoallelic autosomal	Altered gene product sequence	Missense (S358L)
*Rare familial disorder with ARVC*					
*JUP* (ND)	Strong	AR	Biallelic autosomal	Altered gene product sequence	Frameshift variant NMD escaping; Missense; inframe deletion

^a^Gene-disease validity—ClinGen classification (https://clinicalgenome.org/)

^b^PLN-related intrinsic cardiomyopathy is also recorded under HCM in Additional file 3: Table S1

^c^Typified by incomplete penetrance

^d^Typified by age-related onset

^e^NMD truncating = truncating variants nonsense mediated decay (NMD) triggering: frameshift, stop gained, splice acceptor/donor, splice region/intronic variants with proven effect on splicing

*AD* Autosomal dominant, *AR* Autosomal recessive; *indels*, insertions or deletions, *IC* Intrinsic cardiomyopathy, *ND* Naxos disease, *NMD* nonsense-mediated decay, *PSI* Percent spliced in (only variants in TTN that are in or impact exons constitutively expressed in both major adult cardiac isoforms (PSI > 0.9) should be prioritised)

**Table 2:** Structured representation of data from curation of channelopathy gene-disease pairs (LQTS, SQTS, CPVT, BrS)

**Channelopathy**					
**Gene**	**Gene-disease validity**^**a**^	**Inheritance**	**Allelic requirement**	**Disease-associated variant consequence**	**Variant classes reported with evidence of pathogenicity**
**Long QT syndrome (LQTS)**					
*Familial long QT syndrome*					
*KCNQ1*	Definitive	AD; AR^b^	Monoallelic autosomal; biallelic autosomal	Decreased gene product level; altered gene product sequence	Missense; inframe indels; NMD truncating^d^; structural variants (multi exon deletions and a duplication)
*KCNH2*	Definitive	AD^b^	Monoallelic autosomal	Decreased gene product level; altered gene product sequence	Missense; inframe indels; NMD truncating^d^; structural variants (whole exon deletions and duplications)
*SCN5A*	Definitive	AD^b^	Monoallelic autosomal	Altered gene product sequence	Missense; inframe indels
*Long QT Syndrome with atypical features*					
*CALM1*	Definitive	AD^c^	Monoallelic autosomal	Altered gene product sequence	Missense
*CALM2*	Definitive	AD^c^	Monoallelic autosomal	Altered gene product sequence	Missense
*CALM3*	Definitive	AD^c^	Monoallelic autosomal	Altered gene product sequence	Missense
*TRDN*	Strong	AR^c^	Biallelic autosomal	Absent gene product level; altered gene product sequence	NMD truncating^d^; missense
*Syndrome with QT prolongation and cardiac arrhythmias*					
*KCNQ1* (JLNS)	Definitive	AR	Biallelic autosomal	Absent gene product level; altered gene product sequence	Missense; inframe indels; NMD truncating^d^; structural variants (whole exon deletions); complex rearrangements
*KCNE1* (JLNS)	Moderate	AR	Biallelic autosomal	Altered gene product sequence	Missense; inframe indels; stop gained NMD escaping
*KCNJ2* (ATS)	Definitive	AD	Monoallelic autosomal	Altered gene product sequence	Missense; inframe indels; stop gained NMD escaping
*CACNA1C* (TS)	Definitive	AD^c^	Monoallelic autosomal	Altered gene product sequence	Missense
**Brugada Syndrome (BrS)**					
*SCN5A*	Definitive	AD^b^	Monoallelic autosomal	Decreased gene product level; altered gene product sequence	Missense; inframe indels; NMD truncating^d^
**Catecholaminergic polymorphic ventricular tachycardiac (CPVT)**					
*Classic CPVT phenotype*					
*RYR2*	Definitive	AD^b^	Monoallelic autosomal	Altered gene product sequence	Missense; structural variants (exon 3 deletion)
*CASQ2*	Definitive	AR	Biallelic autosomal	Absent gene product level; altered gene product sequence	Missense; NMD truncating^d^
*CASQ2*	Moderate	AD^b^	Monoallelic autosomal	Decreased gene product level; altered gene product sequence	Missense; NMD truncating^d^
*Atypical CPVT Phenotype*					
*CALM1*	Moderate	AD^c^	Monoallelic autosomal	Altered gene product sequence	Missense
*CALM2*	Moderate	AD^c^	Monoallelic autosomal	Altered gene product sequence	Missense
*CALM3*	Moderate	AD^c^	Monoallelic autosomal	Altered gene product sequence	Missense
*TRDN*	Definitive	AR	Biallelic autosomal	Absent gene product level; altered gene product sequence	Missense; NMD truncating^d^; structural variants (exon 2 deletion)
*TECRL*	Definitive	AR	Biallelic autosomal	Absent gene product level; altered gene product sequence	Missense; NMD truncating^d^; structural variants (exon 2 deletion)
**Short QT syndrome (SQTS)**					
*Classic SQTS*					
*KCNH2*	Definitive	AD	Monoallelic autosomal	Altered gene product sequence	Missense
*KCNQ1*	Strong	AD^c^	Monoallelic autosomal	Altered gene product sequence	Missense
*SLC4A3*	Moderate	AD	Monoallelic autosomal	Altered gene product sequence	Missense
*KCNJ2*	Moderate	AD	Monoallelic autosomal	Altered gene product sequence	Missense
*Syndrome including shortened QT and cardiac arrhythmias*					
*SLC22A5* (PSCD)	Definitive	AR	Biallelic autosomal	Altered gene product sequence	Missense

^a^Gene-disease validity—ClinGen classification (https://clinicalgenome.org/)

^b^Typified by incomplete penetrance

^c^Typically de novo

*AD* Autosomal dominant, *AR* Autosomal recessive, *ATS* Andersen-Tawil Syndrome, *indels* Insertions or deletions, *JLNS*, Jervell and Lange-Nielsen Syndrome, *NMD* Nonsense-mediated decay, *PSCD* Primary systemic carnitine deficiency, *TS* Timothy Syndrome

^d^NMD truncating = truncating variants nonsense-mediated decay (NMD) triggering: frameshift, stop gained, splice acceptor/donor, splice region/intronic variants with proven effect on splicing

Inheritance, allelic requirement, and disease-associated variant consequences (as a proxy for disease mechanism) are described using previously agreed standardised terms developed by the GenCC [9]. These terms are formalised in the sequence ontology (SO) [21] and human phenotype ontology (HPO) [22]. Briefly, since the precise disease mechanism is not always known, six high-level variant-consequence terms are used to describe disease-associated variant consequences. These are assigned depending on which variant classes are associated with disease (see Tables 2 and 3 in Roberts et al. [9]). As examples, “decreased gene product level” [SO:0002316] is used when disease is caused by variants that decrease the level or amount of gene product produced (e.g. variants leading to premature termination codons (PTCs) that trigger nonsense mediated decay (NMD), and gene deletions) and “altered gene product sequence” [SO:0002318] is used for non-truncating variants that instead alter the sequence of the gene product such as the amino acid sequence of a protein (e.g. missense variants, inframe insertions or deletions (indels), PTCs predicted to escape NMD, and stop loss). Variants producing PTCs are often referred to as “loss of function (LoF)” variants, but a PTC could lead to LoF, gain of function (GoF) through loss of a terminal regulatory region, or dominant negative effect. Similarly missense variants can cause GoF, LoF, or dominant negative effects. Using known pathogenic variant classes to describe which consequences, at a sequence level, have been associated with disease allows prediction of which other variant classes may be pathogenic whilst recognising that the downstream mechanisms following a particular sequence consequence can be diverse [9]. More than one disease-associated variant consequence term can be used for each gene-disease pair.

Evidence was collected primarily from published, peer-reviewed literature, but also publicly accessible resources such as ClinGen [13] and variant databases (e.g. ClinVar [23]). Building on the previous work by ClinGen GCEPs to determine gene-disease validity, each gene-disease pair was analysed by an individual curator following a standard operating procedure for determining inheritance and disease-associated variant consequences (see Additional file 1). Curation results were then reviewed by panels of international experts (clinicians and scientists) drawn from the relevant disease area.

### Development of CardiacG2P

A structured representation of the resulting data is available in Additional files 2, and 3 and also through G2P (https://www.ebi.ac.uk/gene2phenotype/downloads), which is also searchable through the GenCC portal [8].

For each curation entry, a gene or locus is linked to a disease via a disease-associated variant consequence (as a proxy for disease mechanism) and allelic requirement. Additional information including a confidence category of gene-disease validity (as previously assigned by ClinGen), a narrative summary describing key messages from the expert review, and relevant publication identifiers is also stored.

Unless specifically mentioned, genes previously curated for validity by ClinGen, but not classified as “Definitive” or “Strong” for cardiac disease are included on the panel for completeness. The panel reports the gene-disease validity classification (e.g. “Limited” evidence), but does not speculate on inheritance and mechanism terms where the gene-disease relationship is not established (for information, see the current version of the ClinGen gene-disease validity SOP [24]).

### Validating CardiacG2P

**Fig. 2:**
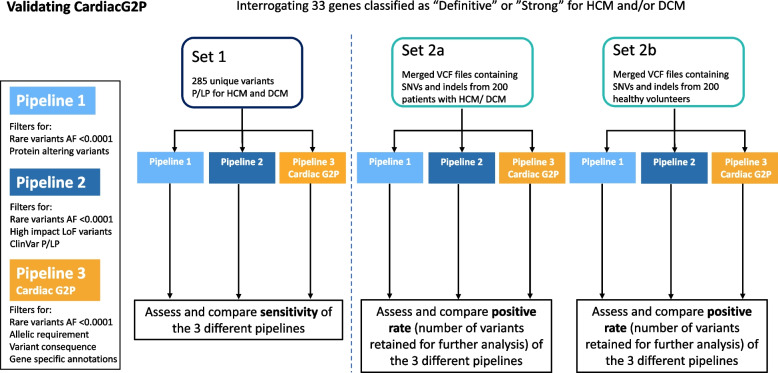
Validating CardiacG2P. Two generic variant prioritisation pipelines (pipelines 1 and 2) were compared to CardiacG2P (pipeline 3). All 3 pipelines interrogate the same gene list which includes 21 HCM and 12 DCM genes. *Pipeline 1*: filtered rare (gnomAD global allele frequency (AF) <0.0001) AND protein-altering variants. *Pipeline 2*: filtered rare (AF <0.0001) AND ((high impact variants (e.g. stop gained, frameshift) AND high confidence by LOFTEE (VEP plugin) LoF variants) OR ClinVar P/LP variants). *CardiacG2P (pipeline 3)*: filtered rare variants (AF <0.0001) and incorporates allelic requirement, variant consequence, and gene-specific annotations of a restricted repertoire of pathogenic alleles appropriate for the disease under interrogation—e.g. restricted variant classes, specific variants, or restricted regions of the protein. *Set 1*: contains 285 unique variants identified and classified as P/LP for HCM or DCM by a specialist NHS cardiovascular genetics lab. A VCF file with these variants was created, annotated by VEP, and filtered according to the 3 pipelines. Sensitivity (number of P/LP variants retained) was assessed. *Set 2a*: is a merged VCF file with SNVs and indels from 200 patients with HCM or DCM. *Set2b*: is a merged VCF file with SNVs and indels from 200 healthy volunteers. Set2a and 2b were separately annotated by VEP and filtered according to the 3 pipelines. Positive rate (the number of variants retained for further analysis) was assessed. AF, allele frequency; DCM, dilated cardiomyopathy; HCM, hypertrophic cardiomyopathy; indels, insertion or deletion variants; LoF, loss of function; P/LP, pathogenic/likely pathogenic; SNVs, single nucleotide variants; VCF, variant call format; VEP, variant effect predictor

We evaluated the utility of CardiacG2P by comparing a variant prioritisation pipeline incorporating data from this structured resource against two alternative generic approaches available to an analyst without disease-specific expertise (see Fig. 2). All three pipelines interrogate the same gene list which includes the 21 HCM and 12 DCM genes evaluated here.

- *Pipeline 1*: Generic bioinformatics analysis pipeline with 3-step filtering approach: filtering on gene symbol (for 33 gene-disease relationships classified by ClinGen as “Strong” or “Definitive” for HCM and/or DCM), retaining only rare variants (gnomAD [25] global allele frequency <0.0001), retaining only protein-altering variants (PAVs).
- *Pipeline 2*: Generic bioinformatics analysis pipeline with 4-step filtering approach: on gene symbol, retaining only rare variants (gnomAD global allele frequency <0.0001), retaining variants that are either high impact (i.e. protein truncating variants (e.g. stop gained, frameshift) AND predicted to result in loss of function with high confidence by LOFTEE [25], a VEP plugin), OR that are previously classified in ClinVar [23] as P/LP (as annotated by VEP [11] version 104).
- *Pipeline 3 (Cardiac G2P)*: Using CardiacG2P dataset, variants were filtered: on gene symbol, retaining only rare variants (gnomAD global allele frequency <0.0001), and with allelic requirement, variant consequence, and gene-specific annotations of a restricted repertoire of pathogenic alleles all appropriate for the disease under interrogation—e.g. restricted variant classes, specific variants, or restricted regions of the protein. Specific examples include removing all *TTN* missense variants apart from three with segregation evidence. In addition for *MYBPC3,* all intronic variants were retained given recent work identifying more deeply intronic variants associated with disease. This information is available in either the restricted repertoire of pathogenic variants or narrative summaries.

To compare these different approaches, two test sets of data were generated (see Fig. 2). Information on filtering steps is also available in Additional file 3: Tables S2–S4.

#### Set 1: To assess sensitivity

Set 1 contains 285 unique gold-standard true positive variants classified as P/LP for HCM and DCM in the last 3 years by the Clinical Genetics & Genomics Laboratory of the NHS Genomic Medicine Service South-East Genomics Laboratory Hub at the Royal Brompton Hospital, London, which is one of 4 NHS England specialist cardiovascular genetics labs. These variants were identified using a custom gene panel using Agilent SureSelect QXT library preparation sequenced on Illumina MiSeq or NextSeq platforms. All variants were evaluated following guidelines produced by the ACMG/AMP [26] and the Association for Clinical Genomic Science (ACGS) [27] using an in-house validated pipeline.

For this study, a variant call format (VCF) file was created using these variants, then annotated using VEP [11] version 104, and filtered according to the 3 pipelines. We compared the number of P/LP variants retained by each of the 3 methods.

#### Set 2: To assess the positive rate—the number of variants retained for further analysis

Set 2a contains data from 200 patients with cardiomyopathy (either HCM or DCM) from the Royal Brompton & Harefield Hospitals Cardiovascular Research Biobank. Set 2b contains data from 200 healthy volunteers recruited for the digital heart project [28]. Participants provided written informed consent, and the research had ethics committee approval. No individual patient data is reported. The GRCh37 reference genome assembly (Ensembl/GENCODE version 19) was used for sequencing and analysis. Details of the sequencing panels and platforms and the bioinformatics pipelines used for variant calling are previously reported [29]. Briefly, samples were sequenced using the Illumina TruSight Cardio Sequencing Kit, which includes 174 genes reported as associated with ICCs, on the Illumina MiSeq and NextSeq platforms. Targeted DNA libraries were prepared according to manufacturers’ protocols before performing paired-end sequencing. For this study, merged VCF files containing single nucleotide variants (SNVs), and insertion or deletion variants were annotated using VEP version 104 and filtered according to the 3 pipelines described above.

Since it is not possible to define a gold-standard classification for these variants that does not incorporate the same expert knowledge captured in CardiacG2P (except potentially for a very small number of variants with orthogonal segregation data), we report the total number of variants retained by each of the three methods (the positive rate), rather than positive predictive value. This is indicative of the analytical burden for a diagnostic laboratory manually interpreting variants of interest retained by a filtering pipeline. We have included a healthy cohort to represent the potential analytical burden of secondary findings.

## Results

### Inheritance and disease-associated variant consequences in established ICC genes

Forty cardiomyopathy gene-disease pairs (22 for HCM, 12 for DCM, and 6 for ARVC; overall 33 unique genes) were analysed for inheritance pattern, allelic requirement, disease-associated variant consequences, and variant classes reported with evidence of pathogenicity. These are presented in Table 1 (typical HCM, DCM, and ARVC) and Additional file 3: Table S1 (syndromic disorders that include HCM where LVH may be a presenting feature). Twenty-five channelopathy gene-disease pairs (11 for LQTS, 1 for BrS, 8 for CPVT, and 5 for SQTS; overall 15 unique genes) are presented in Table 2. Narrative summaries accompany each gene-disease pair, with content including relevant transcripts, specific pathogenic variants, mutational hotspots, phenotype notes, and other important information raised during the expert panel reviews and discussion (see Additional file 2 or Additional file 3: Tables S6–S7).

### Cardiomyopathy

Cardiomyopathy genes are predominately characterised by autosomal dominant inheritance with incomplete penetrance. However, 3/6 ARVC genes demonstrate both autosomal dominant and recessive inheritance; *JUP*-related Naxos disease (a syndrome characterised by ARVC, woolly hair, and palmoplantar keratoderma) is exclusively inherited in an autosomal recessive manner, and 3/14 syndromic HCM genes (*FHL1, GLA* and *LAMP2*) are X-linked.

Importantly, only one of the eight core sarcomere-encoding HCM-associated genes (*MYBPC3*) causes disease through haploinsufficiency. LoF is not an established mechanism for the other 7 core HCM genes (as listed in Table 1) and NMD-competent PTCs are not known to be pathogenic. Instead, missense variants and variants predicted to escape NMD leading to an altered gene product sequence rather than decreased gene product level should be prioritised. This is also the case for 8/14 syndromic HCM (*CACNA1C, FLNC, PRKAG2, PTPN11* (Noonan), *PTPN11* (Noonan syndrome with multiple lentigines), *RAF1, RIT1, TTR*), 3/12 DCM (*DES, TNNC1* and *TNNT2*), and 2/6 ARVC (*JUP, TMEM43*) gene-disease pairs.

Additional useful information for variant filtering is captured in individual narrative summaries. For example, for *TTN*-related DCM, only PTCs that are in exons constitutively expressed in both major adult cardiac isoforms (PSI > 0.9) should be prioritised [28, 30, 31]. Very few pathogenic missense variants in *TTN-*related DCM have been identified: to our knowledge, there are only three reported with segregation evidence [32–34]. Individually rare missense variants in *TTN* are collectively extremely common in the population (>50%, depending on allele frequency cut-off), and there are seldom established approaches to prioritise these in the absence of an informative pedigree. There are instances where evidence for disease comes primarily from one variant class such as missense variants only in *MYL2, MYL3,* and *TPM1*-related HCM, or from a single well-characterised variant, such as *TMEM43*-related ARVC and the founder missense variant NM_024334.3(TMEM43) c.1073C>T (p.S358L) [35]. Pathogenicity of other variant classes, or indeed other missense variants, for *TMEM43* is not established and this should guide the interpretation of variants in these gene-disease relationships.

For some gene-disease relationships, there are gene regions where there is a high confidence for pathogenicity, for example exon 9 in *RBM20-*related DCM (RS motif, amino acids 634-638). Other examples of mutational hotspots are referenced in individual curations.

### Channelopathy

The channelopathy genes are predominately characterised by autosomal dominant inheritance, though 7/25 gene-disease pairs demonstrate autosomal recessive inheritance.

For 7/11 LQTS, 4/7 CPVT and 5/5 SQTS, disease is due to altered gene product sequence and not a decrease in gene product level. For these gene-disease relationships, it is missense variants and other non-truncating variants that should be prioritised and assessed for pathogenicity.

Many of the channelopathy genes are implicated in more than one phenotype, or overlapping phenotypes; 25 gene-disease relationships are evaluated here but only 15 unique genes. Importantly, for several genes, distinct variant classes drive different phenotypes through distinct mechanisms. As an example, both PTCs and missense variants leading to LoF of *KCNQ1* are associated with LQTS and Jervell Lange-Nielsen syndrome. In contrast, almost all evidence for *KCNQ1* as a cause of SQTS is derived from a single missense variant, NM_000218.3(KCNQ1):c.421G>A (p.Val141Met), and functional studies in cell models have confirmed GoF as the mechanism [36, 37]. Similarly, both PTCs and non-truncating variants leading to LoF of *SCN5A* are associated with BrS, whereas *SCN5A*-related LQTS is caused by pathogenic missense variants and inframe indels leading to GoF.

For certain gene-disease pairs, there are gene regions where there is a higher confidence for pathogenicity such as, for non-truncating variants, the transmembrane regions and C-terminus domains for *KCNQ1*-related LQTS [38, 39], and the ion channel transmembrane regions and specific N-terminus and C-terminus domains for *KCNH2*-related LQTS [39]. There are other examples of mutational hotspots referenced in individual curations (see Additional file 2 or Additional file 3: Tables S6–S7).

### CardiacG2P reduces the number of variants prioritised, without compromising sensitivity to detect true positives

#### Assessing sensitivity

**Fig. 3:**
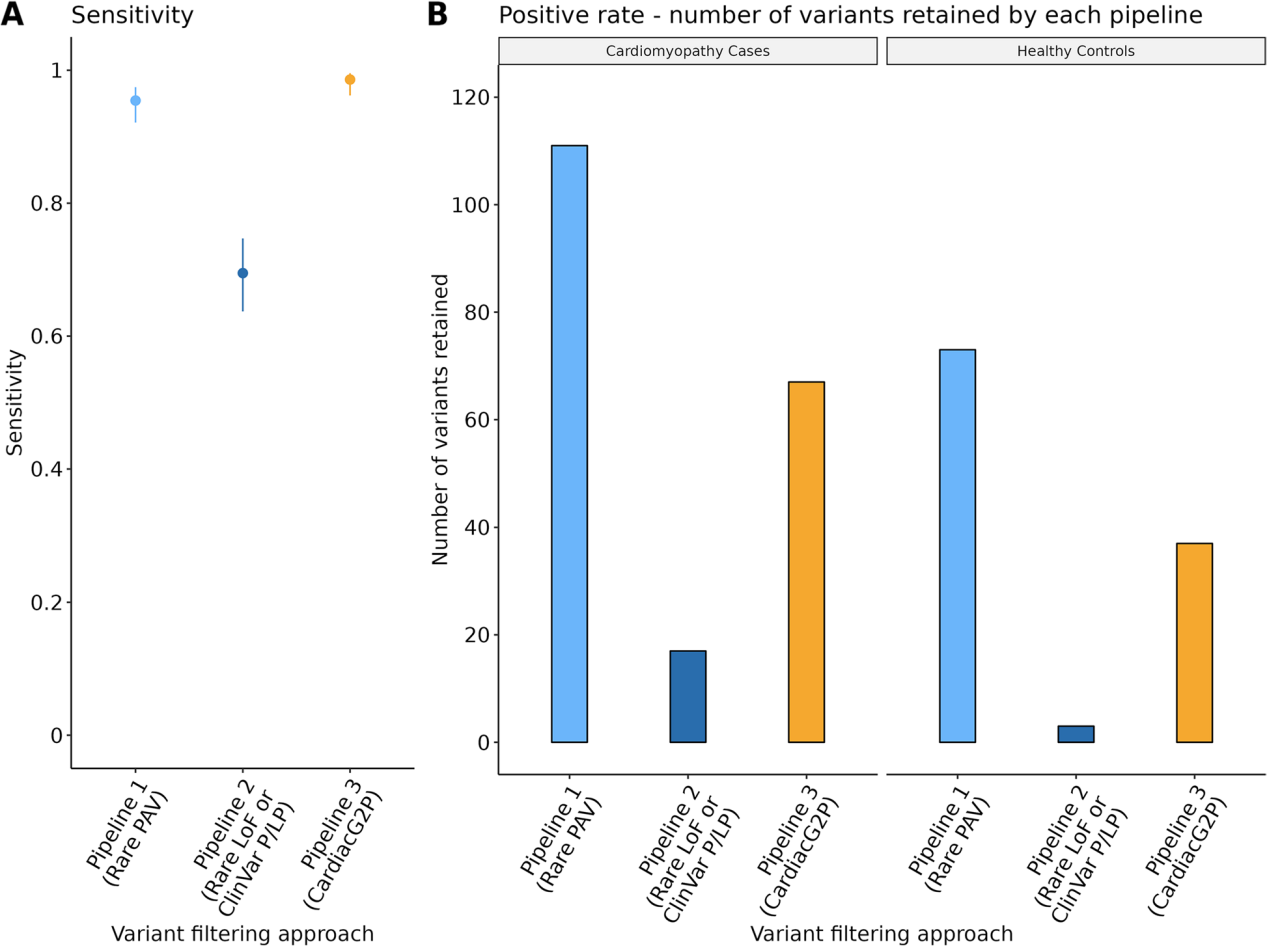
A variant prioritisation approach that incorporates structured data representing disease mechanisms and allelic requirement for specific gene-disease pairs (CardiacG2P) outperforms other scalable variant-prioritisation approaches.** A** Comparison of the sensitivity of 3 variant filtering approaches to prioritise 285 variants classified as pathogenic/likely pathogenic (P/LP) for hypertrophic cardiomyopathy (HCM) and dilated cardiomyopathy (DCM). Error bars = 95% confidence intervals (CI). Pipeline 1 (light blue) prioritises all rare protein-altering variants (PAV), sensitivity 0.95, 95% CI [0.92, 0.97]. Pipeline 2 (dark blue) prioritises all rare loss of function (LoF) variants, and those classified as P/LP by ClinVar, sensitivity 0.70, 95% CI [0.64, 0.75]. Pipeline 3 (orange) prioritises variant classes according to specific characteristics of each gene-disease pair (CardiacG2P), sensitivity 0.99, 95% CI [0.96, 1.0]. CardiacG2P has a higher sensitivity when compared to Pipeline 1, *P*_Fisher_ = 0.046 and Pipeline 2, *P*_Fisher_ ≤0.0001. **B** The positive rate (number of variants retained) by 3 variant-filtering approaches for cardiomyopathy cases (left panel), using a dataset of 5681 unique variants from 200 individuals with confirmed HCM/DCM, and healthy controls (right panel), using a dataset of 6060 unique variants from 200 healthy individuals. Pipeline 1 (light blue), filtering for rare PAV; Pipeline 2 (dark blue), filtering for rare LoF variants or those classified as P/LP by ClinVar. Pipeline 3 (orange), filtering using CardiacG2P. CardiacG2P demonstrated more efficient variant prioritisation compared to Pipeline 1 in both the disease cohort (*P*_Fisher_ = 0.001) and healthy controls (*P*_Fisher_ ≤0.001)

We assessed variant filtering using the CardiacG2P dataset for the identification of known P/LP variants previously classified by the cardiovascular laboratory of the NHS Genomic Medicine Service South-East Genomics Laboratory Hub at the Royal Brompton Hospital, London. A total of 285 P/LP variants in 16 HCM/DCM genes were used to assess the performance of the CardiacG2P dataset compared to two other generic pipelines (see Fig. 3A). CardiacG2P correctly identified 281/285 variants, a sensitivity of 98.6%. This was superior to both alternative approaches (pipeline 1, 272/285, sensitivity 95.4%, *P*_Fisher_=0.046; pipeline 2, 198/285, 69.5%, *P*_Fisher_ ≤ 0.0001). Four variants were not retained by using the CardiacG2P dataset. These comprised 1 *TTN* missense variant and 2 intronic and 1 synonymous variant in *LMNA*. All four of these variants were classified as P/LP by the clinical laboratory due to impacts on splicing, so the limited sensitivity is due to an incomplete upstream annotation of the variant consequence, rather than an “error” in downstream filtering.

#### Assessing variant prioritisation—the number of variants retained for further analysis

We compared the number of variants retained by the 3 pipeline filters to assess the positive rate of each approach (see Fig. 3B). A pipeline with a high positive rate requires more downstream human effort for final variant adjudication.

First, we compared sequencing data (5681 unique variants) from 200 individuals with a confirmed diagnosis of HCM or DCM. CardiacG2P prioritised 67 variants, pipeline 1 prioritised 111 variants, and pipeline 2 prioritised 17.

Since the cardiomyopathy cohort would be very substantially enriched for true positives, we also assessed the positive rate in a healthy cohort, indicative of variants that may require follow-up during opportunistic screening for secondary findings. 6060 unique variants found in 200 healthy volunteers were analysed by each pipeline, with CardiacG2P prioritising 37 variants, pipeline 1 prioritising 73 variants, and pipeline 2 prioritising 3 variants.

Pipeline 2 prioritises the fewest variants in both contexts (17/5681 and 3/6060 respectively). This is to be expected as it filters on only high-impact LoF variants or variants classified as P/LP by ClinVar. However, this method also demonstrated the lowest sensitivity for P/LP variants (69.5%), because LoF is not a known mechanism for many of the ICC genes and any pathogenic missense or other non-truncating variants will be wrongly discarded by this method. In the disease cohort, compared to pipeline 1 which retains all PAVs, CardiacG2P demonstrated more efficient variant prioritisation retaining significantly fewer variants (*P*_Fisher_ = 0.001). In the healthy cohort, where we would expect a higher number of false-positive variants to be prioritised, CardiacG2P retained half the number of variants compared to pipeline 1 (37 vs. 73 variants, *P*_Fisher_ ≤ 0.001). CardiacG2P also maintained the highest sensitivity of all 3 pipelines at 98.6%.

## Discussion

Accurate variant classification in ICC genes requires robust strength of a gene-disease relationship and knowledge of inheritance pattern, disease mechanism, and pathogenic variant classes [40]. The literature is constantly expanding with newly reported variants and re-evaluations of historical variant classifications. In ClinVar alone, there are over 1 million variants submitted. Over 49,000 have conflicting interpretations and others are submitted under multiple phenotypes making the relevant disease for the variant classification unclear. Variant classification is expanding beyond laboratories with long-standing interest and expertise in cardiovascular genetics. The ACMG secondary findings list means that others will need to rapidly acquire proficiency in reporting variants in CV genes. The AHA has recently published guidance and a framework to aid the interpretation and clinical application of variants in monogenic cardiovascular disease genes [4]. To assist this process, we have curated the mode of inheritance, allelic requirement, and disease-associated variant consequences, for 65 ClinGen-curated ICC gene-disease pairs (48 unique genes), and following review by multidisciplinary expert panels, present this information as a publicly available structured dataset both here and via CardiacG2P (https://www.ebi.ac.uk/gene2phenotype/downloads), to aid variant analysis. This dataset is compatible with the existing G2P plugin for the widely used Ensembl Variant Effect Predictor.

Overall, for 36/65 gene-disease relationships, the disease is due to altered gene product sequence, not a decrease in gene product level. Therefore, for over 50% of the ICC genes evaluated here, current data cautions against a default prioritisation of predicted protein-truncating variants as pathogenic, with LoF as a presumed mechanism. The majority of the ICC genes are characterised by autosomal dominant inheritance with incomplete penetrance; however, there are notable examples of autosomal recessive and X-linked inheritance and more fully penetrant variants.

As well as the structured data, we have included narrative summaries to capture key notes that arose during evidence collection and expert discussion that may also aid variant filtering and interpretation. Throughout these discussions, several themes that relate to all the ICC genes emerged. It is widely accepted that ICC genes often display incomplete penetrance; however, given that most penetrance estimates have been made using cases [41], expert opinion and emerging evidence agree that overall penetrance may be lower than previously reported. This is particularly relevant and should be considered when assessing patients who have a pathogenic variant identified as a secondary finding outside of families with known disease [41, 42].

There are many examples of autosomal dominant ICC gene-disease relationships where compound heterozygous and homozygous variants, or variants in more than 1 known disease gene, are also reported. Approximately 10% of genotype-positive LQTS patients have >1 pathogenic variant in ≥1 LQTS-related gene [43, 44]. There was debate amongst the expert panel on how this should be recorded. In those instances where phenotypic features of people with biallelic variants are truly different to those with monoallelic variants (e.g. Jervell Lange-Nielsen Syndrome), this may represent true autosomal recessive or digenic inheritance and should be recorded as such. However, it was recognised that for many of the ICC genes, disease severity and penetrance are often the main distinguishing features between monoallelic and biallelic disease. In this circumstance, autosomal dominant inheritance is recorded with further information in the narrative summary acknowledging that if a second P/LP variant is identified, the disease often appears to be more penetrant and more severe [45–48] and can even lead to neonatal lethality.

It is important to interpret variants in the context of a gene-disease relationship rather than in the gene alone [49]. There are several ICC genes implicated in more than one phenotype. For some, distinct mechanisms drive different diseases, e.g. *MYH7*-related HCM and *MYH7*-related DCM. Although both are caused primarily by missense variants in *MYH7* altering the gene product sequence, distinct alleles have opposing effects on sarcomere force generation and drive different phenotypes [50, 51]. In contrast, although *DSP* is also associated with multiple phenotypes (including DCM, DCM with cutaneous features, ARVC, and Carvajal syndrome), these are overlapping and it does not appear that distinct mechanisms drive different presentations. Similarly, although the phenotype most frequently shown by patients with *CALM* pathogenic variants is LQTS, others display CPVT and sudden unexplained death and some *CALM* variants have been associated with both LQTS and CPVT, without evidence of distinct mechanisms underlying different phenotypic manifestations [49, 52].

Here we have evaluated CardiacG2P as a first-tier variant filter. This variant consequence and allelic requirement-aware approach increase the efficiency of variant prioritisation, without compromising on sensitivity, in comparison to two generic bioinformatic filtering pipelines (see Fig. 3). CardiacG2P retains significantly fewer variants than a pipeline where all PAVs are prioritised. The difference between CardiacG2P and the generic pipelines is even more marked in a healthy cohort, highlighting benefits in reducing the analytical burden of assessing secondary findings. Further refinement is also possible using additional variant information stored in the narrative summaries. CardiacG2P correctly identified 281/285 previously classified P/LP variants. The four variants that were not retained comprised 1 *TTN* missense variant and 2 intronic and 1 synonymous variant in *LMNA*. All 4 variants were predicted to have a significant impact on splicing by SpliceAI [53]. Functional data is available to support the splicing effect of 2 of the *LMNA* variants. The *TTN* missense variant has been detected in 4 in-house DCM patients before. CardiacG2P filters are based on the consequence assigned by VEP, and upstream annotation by VEP had not recorded these 4 variants as impacting splicing. Improvements in the prediction of variant consequence, especially for variants impacting splicing, will allow these to be retained. While our framework recognises that some intronic or coding variants can impact splicing, it is not an expected consequence for the vast majority of such variants and therefore these will not be routinely retained. Rarely there will be instances where pathogenic variants are filtered by G2P if the upstream consequence annotation is incomplete or incorrect, so we must caution against simply discarding all non-prioritised variants and must continue to improve tools for variant consequence annotation. In the meantime, utilising tools such as SpliceAI and filtering on known P/LP variants in ClinVar will improve the identification of variants impacting splicing and the sensitivity of variant filtering pipelines.

We recognise the limitations of using relatively small numbers of variants and patients from a single site for our comparison of CardiacG2P to other methods. We also acknowledge we have compared CardiacG2P to two generic pipelines here and not a clinical diagnostic pipeline. However, we maintain that many clinical laboratories not specialising in cardiovascular disease will not have the expert knowledge collated here easily accessible.

As our knowledge of genes and specific variants contributing to ICCs expands, it is possible to update the CardiacG2P dataset dynamically and subsequently include new information in the VEP G2Pplugin.

## Conclusions

As variant reporting moves away from labs with expertise in certain disease areas, it is vital that accurate variant classifications are maintained. Here, we present evidenced-based inheritance and variant consequence curations for robustly associated ICC genes with the benefit of expert review and opinion. We present this data for the first time in a structured format using new standardised terminology. This dataset is a publicly available resource, CardiacG2P, and we have demonstrated here its utility in the filtering of genomic variants in ICC genes.

### Supplementary Information

**Additional file 1.** Standard operating procedure for gene-disease curations. This document provides a template and standard operating procedure for the curation of inheritance, allelic requirement and disease mechanism for gene-disease pairs already curated by ClinGen using standardised terminology.

**Additional file 2.** Inheritance and mechanism curation summaries for all gene-disease pairs. Data from individual gene-disease pair curations presented in individual tables with a narrative summary describing key messages from the expert review with relevant publication identifiers.

**Additional file 3:** **Table S1.** A table showing the curation of syndromic forms of (hypertrophic) cardiomyopathy that can have isolated left ventricular hypertrophy as the presenting feature: structured representation of inheritance, allelic requirement, disease-associated variant consequence, and variant classes reported with evidence of pathogenicity for each gene-disease pair. **Tables S2–S5.** Details of the filtering process of each pipeline for the 3 datasets (**Table S2** - Set 1, **Table S3** - Set2a and **Table S4** -Set2b). Details of the demographics of the cohorts used in Set2a and Set2b are available in **Table S5.** **Tables S6–S8.** The same information that is presented in Additional File 2 is included here in xls format. **Table S6**. (CardiacG2P) includes a structured representation of inheritance and mechanism data for all curated gene-disease pairs. In addition this also includes information for 7 genes related to a syndrome where LVH is seen only with overt syndromic features. **Table S7**. (Narr_sum) has narrative summaries for each gene-disease pair as plain free text. **Table S8**. (Other_limited) is a list of gene-disease pairs where there is no established relationship (gene disease validity assertion from ClinGen); these are included for completeness.

## Data Availability

All data generated during this study are included in this published article. For convenience, a structured representation of the results is also available online through (i) G2P (https://www.ebi.ac.uk/gene2phenotype/downloads), which is also searchable through the GenCC portal (https://thegencc.org/), (ii) a publicly accessible repository in GitHub: https://doi.org/10.5281/zenodo.8434146, and (iii) (https://www.cardiodb.org/cardiac_g2p/Cardiac_G2P_Curations.html).

## Abbreviations

ACGS: Association for Clinical Genomic Science
ACMG SF V3.1: American College of Medical Genetics and Genomics Secondary Findings list
AD: Autosomal dominant
AHA: American Heart Association
AR: Autosomal recessive
ARVC: Arrhythmogenic right ventricular cardiomyopathy
ATS: Andersen-Tawil Syndrome
BrS: Brugada syndrome
ClinGen: The Clinical Genome Resource
CPVT: Catecholaminergic polymorphic ventricular tachycardia
CV: Cardiovascular
DCM: Dilated cardiomyopathy
ES: Exome sequencing
G2P: Gene2phenotype
GCEPs: Gene Curation Expert Panels
GenCC: Gene Curation Coalition
GoF: Gain of function
GS: Genome sequencing
HCM: Hypertrophic cardiomyopathy
HPO: Human phenotype ontology
IC: Intrinsic cardiomyopathy
ICCs: Inherited cardiac conditions
Indels: Insertions or deletions
JLNS: Jervell and Lange-Nielsen
LoF: Loss of function
LQTS: Long QT syndrome
LVH: Left ventricular hypertrophy
ND: Naxos disease
NMD: Nonsense-mediated decay
P/LP: Pathogenic/likely pathogenic
PAVs: Protein altering variants
PSCD: Primary systemic carnitine deficiency
PSI: Percent spliced in
PTCs: Premature termination codons
SNVs: Single nucleotide variants
SO: Sequence ontology
SQTS: Short QT syndrome
TS: Timothy syndrome
VCF: Variant call format
VEP: Variant Effect Predictor

## References

[1] Musunuru K, Hershberger RE, Day SM, Klinedinst NJ, Landstrom AP, Parikh VN (2020). Genetic testing for inherited cardiovascular diseases: a scientific statement from the american heart association. Circulation.

[2] Hershberger RE, Givertz MM, Ho CY, Judge DP, Kantor PF, McBride KL (2018). Genetic evaluation of cardiomyopathy: a clinical practice resource of the American College of Medical Genetics and Genomics (ACMG). Genet Med.

[3] Wilde AAM, Semsarian C, Márquez MF, Sepehri Shamloo A, Ackerman MJ, Ashley EA (2022). European Heart Rhythm Association (EHRA)/Heart Rhythm Society (HRS)/Asia Pacific Heart Rhythm Society (APHRS)/Latin American Heart Rhythm Society (LAHRS) Expert Consensus Statement on the State of Genetic Testing for Cardiac Diseases. Heart Rhythm.

[4] Landstrom AP, Chahal AA, Ackerman MJ, Cresci S, Milewicz DM, Morris AA (2023). Interpreting incidentally identified variants in genes associated with heritable cardiovascular disease: a scientific statement from the American Heart Association. Circulation.

[5] Miller DT, Lee K, Abul-Husn NS, Amendola LM, Brothers K, Chung WK (2022). ACMG SF v3.1 list for reporting of secondary findings in clinical exome and genome sequencing: a policy statement of the American College of Medical Genetics and Genomics (ACMG). Genet Med..

[6] Green RC, Berg JS, Grody WW, Kalia SS, Korf BR, Martin CL (2013). ACMG Recommendations for Reporting of Incidental Findings in Clinical Exome and Genome Sequencing. Genet Med.

[7] DiStefano MT, Goehringer S, Babb L, Alkuraya FS, Amberger J, Amin M (2022). The Gene Curation Coalition: a global effort to harmonize gene-disease evidence resources. Genet Med.

[8] DiStefano MT, Goehringer S, Babb L, Alkuraya FS, Amberger J, Amin M, et al. The GenCC database. https://search.thegencc.org/ . Accessed 3rd April 2022.

[9] Roberts AM, DiStefano MT, Rooney Riggs E, Josephs KS, Alkuraya FS, Amberger J (2023). Towards robust clinical genome interpretation: developing a consistent terminology to characterize disease-gene relationships - allelic requirement, inheritance modes and disease mechanisms. MedRxiv.

[10] Thormann A, Halachev M, McLaren W, Moore DJ, Svinti V, Campbell A (2019). Flexible and scalable diagnostic filtering of genomic variants using G2P with Ensembl VEP. Nat Commun.

[11] McLaren W, Gil L, Hunt SE, Riat HS, Ritchie GRS, Thormann A (2016). The Ensembl Variant Effect Predictor. Genome Biol.

[12] Lenassi E, Carvalho A, Thormann A, Abrahams L, Arno G, Fletcher T (2023). EyeG2P: an automated variant filtering approach improves efficiency of diagnostic genomic testing for inherited ophthalmic disorders Diagnostics. J Med Genet.

[13] Rehm HL, Berg JS, Brooks LD, Bustamante CD, Evans JP, Landrum MJ (2015). ClinGen — The Clinical Genome Resource. N Engl J Med.

[14] Clinical Genome Resource. Clinical Domain Working Groups. https://clinicalgenome.org/working-groups/clinical-domain/. Accessed 1 Nov 2020.

[15] Adler, Novelli V, Amin AS, Abiusi E, Care M, Nannenberg EA (2020). An international, multicentered, evidence-based reappraisal of genes reported to cause congenital long QT syndrome. Circulation.

[16] Hosseini SM, Kim R, Udupa S, Costain G, Jobling R, Liston E (2018). Reappraisal of reported genes for sudden arrhythmic death: evidence-based evaluation of gene validity for Brugada syndrome. Circulation.

[17] Walsh R, Adler A, Amin AS, Abiusi E, Care M, Bikker H (2021). Evaluation of gene validity for CPVT and short QT syndrome in sudden arrhythmic death. Eur Heart J.

[18] James CA, Jongbloed JDH, Hershberger RE, Morales A, Judge DP, Syrris P (2021). International evidence based reappraisal of genes associated with arrhythmogenic right ventricular cardiomyopathy using the clinical genome resource framework. Circulation.

[19] Ingles J, Goldstein J, Thaxton C, Caleshu C, Corty EW, Crowley SB (2019). Evaluating the clinical validity of hypertrophic cardiomyopathy genes. Circulation.

[20] Jordan E, Peterson L, Ai T, Asatryan B, Bronicki L, Brown E (2021). Evidence-based assessment of genes in dilated cardiomyopathy. Circulation.

[21] Eilbeck K, Lewis SE, Mungall CJ, Yandell M, Stein L, Durbin R (2005). The Sequence Ontology: a tool for the unification of genome annotations. Genome Biol.

[22] Gargano M, Matentzoglu N, Carmody LC, Lewis-Smith D, Vasilevsky NA, Danis D (2020). The Human Phenotype Ontology in 2021. Nucleic Acids Res.

[23] Landrum MJ, Lee JM, Benson M, Brown GR, Chao C, Chitipiralla S, et al. ClinVar: improving access to variant interpretations and supporting evidence. Nucleic Acids Res. 2018;46. 10.1093/nar/gkx1153. 10.1093/nar/gkx1153 PMC5753237 29165669

[24] Clinical Genome Resource. Gene-Disease Validity Training Materials - ClinGen | Clinical Genome Resource. https://clinicalgenome.org/curation-activities/gene-disease-validity/training-materials. Accessed 3 April 2022.

[25] Karczewski KJ, Francioli LC, Tiao G, Cummings BB, Alföldi J, Wang Q (2020). The mutational constraint spectrum quantified from variation in 141,456 humans Genome Aggregation Database Consortium. Nature.

[26] Richards S, Aziz N, Bale S, Bick D, Das S, Gastier-Foster J (2015). Standards and guidelines for the interpretation of sequence variants: a joint consensus recommendation of the American College of Medical Genetics and Genomics and the Association for Molecular Pathology. Genet Med.

[27] Ellard S, Baple EL, Callaway A, Berry I, Forrester N, Turnbull C, et al. ACGS Best Practice Guidelines for Variant Classification in Rare Disease 2020 Recommendations ratified by ACGS Quality Subcommittee on 4 th. 2020; 10.1101/531210.

[28] Roberts AM, Ware JS, Herman DS, Schafer S, Baksi J, Bick AG (2015). Integrated allelic, transcriptional, and phenomic dissection of the cardiac effects of titin truncations in health and disease. Sci Transl Med.

[29] Walsh R, Buchan R, Wilk A, John S, Felkin LE, Thomson KL, et al. Defining the genetic architecture of hypertrophic cardiomyopathy: re-evaluating the role of non-sarcomeric genes. Eur Heart J. 2017; ehw603. 10.1093/eurheartj/ehw603. 10.1093/eurheartj/ehw603 PMC5837460 28082330

[30] Schafer S, de Marvao A, Adami E, Fiedler LR, Ng B, Khin E (2017). Titin truncating variants affect heart function in disease cohorts and the general population. Nat Genet.

[31] Morales A, Kinnamon DD, Jordan E, Platt J, Vatta M, Dorschner MO (2020). Variant interpretation for dilated cardiomyopathy (DCM): refinement of the ACMG/ClinGen Guidelines for the DCM Precision Medicine Study Circulation. Genom Precis Med.

[32] Gerull B, Gramlich M, Atherton J, Mcnabb M, Trombitás K, Sasse-Klaassen S, et al. Mutations of TTN, encoding the giant muscle filament titin, cause familial dilated cardiomyopathy. Nat Genet. 2002;30. 10.1038/ng815. 10.1038/ng815 11788824

[33] Herrero Galán E. Conserved cysteines in titin sustain the mechanical function of cardiomyocytes. 10.1101/2020.09.05.282913.

[34] Hastings R, de Villiers CP, Hooper C, Ormondroyd L, Pagnamenta A, Lise S (2016). Combination of whole genome sequencing, linkage, and functional studies implicates a missense mutation in titin as a cause of autosomal dominant cardiomyopathy with features of left ventricular noncompaction. Circulation.

[35] Merner ND, Hodgkinson KA, Haywood AFM, Connors S, French VM, Drenckhahn JD (2008). Arrhythmogenic right ventricular cardiomyopathy type 5 is a fully penetrant, lethal arrhythmic disorder caused by a missense mutation in the TMEM43 gene. Am J Hum Genet.

[36] Lee HC, Rudy Y, Liang H, Chen CC, Luo CH, Sheu SH (2017). Pro-arrhythmogenic effects of the V141M KCNQ1 mutation in short QT syndrome and its potential therapeutic targets: insights from modeling. J Med Biol Eng.

[37] Hong K, Piper D, Diazvaldecantos A, Brugada J, Oliva A, Burashnikov E (2005). De novo KCNQ1 mutation responsible for atrial fibrillation and short QT syndrome in utero. Cardiovasc Res.

[38] Kapa S, Tester DJ, Salisbury BA, Harris-Kerr C, Pungliya MS, Alders M (2009). Genetic testing for long QT syndrome - distinguishing pathogenic mutations from benign variants. Circulation.

[39] Walsh R, Lahrouchi N, Tadros R, Kyndt F, Glinge C, Postema PG (2021). Enhancing rare variant interpretation in inherited arrhythmias through quantitative analysis of consortium disease cohorts and population controls. Genet Med.

[40] Arbustini E, Behr ER, Carrier L, van Duijn C, Evans P, Favalli V (2022). Interpretation and actionability of genetic variants in cardiomyopathies: a position statement from the European Society of Cardiology Council on cardiovascular genomics. Eur Heart J.

[41] Lorenzini M, Norrish G, Field E, Ochoa JP, Cicerchia M, Akhtar MM (2020). Penetrance of hypertrophic cardiomyopathy in sarcomere protein mutation carriers. J Am Coll Cardiol.

[42] de Marvao A, McGurk KA, Zheng SL, Thanaj M, Bai W, Duan J (2021). Phenotypic expression and outcomes in individuals with rare genetic variants of hypertrophic cardiomyopathy. J Am Coll Cardiol.

[43] Tester DJ, Will ML, Haglund CM, Ackerman MJ (2005). Compendium of cardiac channel mutations in 541 consecutive unrelated patients referred for long QT syndrome genetic testing.

[44] Kapplinger JD, Tester DJ, Salisbury BA, Carr JL, Harris-Kerr C, Pollevick GD (2009). Spectrum and prevalence of mutations from the first 2,500 consecutive unrelated patients referred for the FAMILION® long QT syndrome genetic test. Heart Rhythm.

[45] Bhonsale A, Groeneweg JA, James CA, Dooijes D, Tichnell C, Jongbloed JD H (2015). Impact of genotype on clinical course in arrhythmogenic right ventricular dysplasia/cardiomyopathy-associated mutation carriers. Euro Heart J.

[46] Kolokotronis K, Kühnisch J, Klopocki E, Dartsch J, Rost Simone, Huculak C (2019). Biallelic mutation in MYH7 and MYBPC3 leads to severe cardiomyopathy with left ventricular noncompaction phenotype. Hum Mutat.

[47] Alders M, Bikker H, Christiaans I (2003). Long QT syndrome.

[48] Girolami F, Ho CY, Semsarian C, Baldi M, Will ML, Baldini K (2010). Clinical features and outcome of hypertrophic cardiomyopathy associated with triple sarcomere protein gene mutations. J Am Coll Cardiol.

[49] Thaxton C, Goldstein J, DiStefano M, Wallace K, Witmer PD, Haendel MA (2022). Lumping versus splitting: how to approach defining a disease to enable accurate genomic curation. Cell Genom.

[50] Ujfalusi Z, Vera CD, Mijailovich SM, Svicevic M, Yu EC, Kawana M (2018). Dilated cardiomyopathy myosin mutants have reduced force-generating capacity. J Biol Chem.

[51] Sommese RF, Sung J, Nag S, Sutton S, Deacon JC, Choe E (2013). Molecular consequences of the R453C hypertrophic cardiomyopathy mutation on human β-cardiac myosin motor function. Proc Natl Acad Sci USA.

[52] Crotti L, Spazzolini C, Tester DJ, Ghidoni A, Baruteau AE, Beckmann BM (2019). Calmodulin mutations and life-threatening cardiac arrhythmias: insights from the International Calmodulinopathy Registry. Eur Heart J.

[53] Jaganathan K, Kyriazopoulou Panagiotopoulou S, McRae JF, Darbandi SF, Knowles D, Li YI (2019). Predicting splicing from primary sequence with deep learning. Cell.

